# All-cause mortality of patients with idiopathic pulmonary fibrosis: a nationwide population-based cohort study in Korea

**DOI:** 10.1038/s41598-021-94655-x

**Published:** 2021-07-26

**Authors:** Sung Jun Ko, Sun Mi Choi, Kyung-Do Han, Chang-Hoon Lee, Jinwoo Lee

**Affiliations:** 1 Department of Internal Medicine, Wonkwang University Sanbon Hospital, Gunpo, Republic of Korea; 2 Division of Pulmonary and Critical Care Medicine, Department of Internal Medicine, Seoul National University College of Medicine, Seoul National University Hospital, 101 Daehak-Ro, Jongno-Gu, Seoul, 03080 Republic of Korea; 3 Department of Biostatistics, College of Medicine, The Catholic University of Korea, Seoul, Republic of Korea

**Keywords:** Respiratory tract diseases, Epidemiology

## Abstract

Most epidemiologic studies of patients with idiopathic pulmonary fibrosis (IPF) have been conducted in North America and Europe. Moreover, there are limited data concerning the cause of death and cause-specific mortality rate of IPF patients in population-based studies. We analyzed information from the Korean National Health Insurance Service database from 2006 to 2016. Patients with a diagnosis code of IPF were extracted from the database and we included those who satisfied the narrow definition of IPF diagnosis. Age- and sex-matched controls were randomly selected at a case-to-control rate of 1:3. We included 42,777 patients newly diagnosed with IPF during the study period. Their mean age was 64.6 years, and 65.4% were male. The age-standardized mortality rates were 85.66 (95% confidence interval [CI] 84.45–86.89) per 1000 person-years. The survival rates of IPF patients 1, 2, 3, 5, and 10 years after IPF diagnosis were 84.5%, 77.4%, 71.9%, 62.9%, and 48.4%, respectively. The standardized mortality ratio of IPF patients compared to that of the normal population was 4.66. The leading cause of death in IPF patients was respiratory causes, followed by cancer. Patients with IPF in Korea showed significantly higher mortality compared to the general population.

## Introduction

Idiopathic pulmonary fibrosis (IPF) is the most common idiopathic interstitial pneumonia and is associated with a high mortality rate^1^. While there is evidence of increasing IPF incidence and mortality^2–4^, most epidemiologic data on IPF patients, including mortality, were derived from patients in Europe and North America^5^ and the results are highly variable according to study design and statistical methods used to estimate mortality^2–4,6–12^. The mortality of IPF patients is also variable between ethnicities^2,6,9^. Moreover, the reported mortality of IPF patients in population-based studies has not been compared to that of the general population.

In East Asia, epidemiological data on IPF based on a nationwide population database are scarce. Although two population-based studies were performed in Taiwan and Japan^11,12^, the sample sizes were much smaller than those of studies from Europe and North America. Cohort studies of IPF patients in Korea have investigated the clinical course of IPF; however, such studies may be prone to selection bias and only included a small number of participants and, thus, lack generalizability^13,14^.

Despite the high mortality of IPF patients, there are limited data concerning the causes of death and cause-specific mortality rate of IPF patients in population-based studies^2,6,11^. Along with differences in the mortality rates of IPF patients, there may also be differences in the causes of death across different ethnicities. While the leading cause of death among IPF patients was respiratory causes, followed by ischemic heart disease in the United States^2,6^, the second most frequent cause of death was lung cancer in Japan^11^. To our knowledge, cause-specific mortality rates of IPF patients in population-based studies have not been compared to those of the general population.

One of the main causes of death in IPF patients is respiratory failure due to disease progression which is often inevitable despite advanced care. Except for IPF patients awaiting lung transplantation, mechanical ventilator support for respiratory failure in IPF patients is generally not recommended because of their very high mortality^15–18^. However, the exact proportion of patients with IPF receiving intensive care before their deaths is unknown.

This study investigated the all-cause and cause-specific mortality rates of IPF patients and compared them to the age- and sex-matched general population using a nationwide population-based database. The causes of death and rates of intensive care unit (ICU) admission before death were also assessed.

## Methods

### Study participants

The medical demands of nearly all Korean citizens are mandatorily covered by the National Health Insurance Service (NHIS). From the beginning of full coverage in 1989, the NHIS in Korea has accumulated a tremendous amount of data, including patient diagnosis codes and their claims for medical tests and prescriptions. The NHIS has provided its data to medical researchers for the public interest since the establishment of the database in 2002. A detailed description of the NHIS database has been published elsewhere^19^.

A more narrow definition of IPF was used in this study. Patients with the diagnosis code of IPF (Korean Standard Classification of Disease code J841 or J8418) from January 2006 to December 2016 were initially selected for inclusion in the present study. A narrow definition of IPF was fulfilled after excluding these three conditions. (1) Since the diagnosis of IPF requires multidisciplinary discussion, we excluded patients if the diagnosis of IPF was claimed only in the primary clinics but not in referral hospitals. (2) Patients who did not undergo a pulmonary function test (PFT) within 6 months and chest computed tomography (CT) within 1 year of IPF diagnosis also were excluded. (3) Patients with diagnosis codes of connective tissue diseases (CTDs) were also excluded by their diagnostic codes of CTDs to exclude CTD-associated interstitial lung diseases. To compare the mortality of IPF patients with that of the general population without IPF, age- and sex-matched controls (three times the number of cases) were randomly extracted from the NHIS database. The comorbidities of the patients in the IPF and control groups were collected from their diagnosis codes. Household income and place of residence were also gathered for comparisons between groups. Death certificate data were obtained from the National Statistical Office of Korea to evaluate the survival and causes of death of the study participants.

Claims data of patients admitted to the ICU within 30 days before the date of death were collected. These patients were considered as having received end-of-life care in the ICU.

This study was approved by the Institutional Review Board (IRB) of the Seoul National University Hospital (SNUH) (IRB No. E-1804-046-936). The requirement for informed consent was waived by the IRB of SNUH due to the retrospective design of this study utilizing the NHIS database. All research was performed in accordance with the Declaration of Helsinki concerning the ethical principles for medical research.

### Statistical analysis

The mortality rate was described as the incidence rate per 1000 person-years. Mortality rates were calculated in both male and female individuals in the IPF and control groups. The hazard ratio (HR) of IPF patients compared to that in the control group adjusted for age, sex, presence of comorbidities (ischemic heart disease, stroke, cerebrovascular disease, cancer, diabetes mellitus, hypertension, dyslipidemia, and liver disease), household income, and place of residence was analyzed by Cox regression analysis. The differences in cause-specific mortality rates between IPF patients and the controls were also analyzed. We also evaluated the difference between all-cause mortality of IPF patients and the control group using Kaplan–Meier curve analysis.

To calculate the standardized mortality ratio (SMR) of IPF patients, we compared the observed death events in IPF patients to the expected death events of the general population derived from Korean Statistical Information Service data. We also calculated the SMR in the pre-stratified age groups.

Statistical analysis was performed using R (version 3.2.3, https://www.R-project.org/). All statistical tests were two-sided and differences with *p* < 0.05 were considered statistically significant.

## Results

### Study population

**Figure 1:**
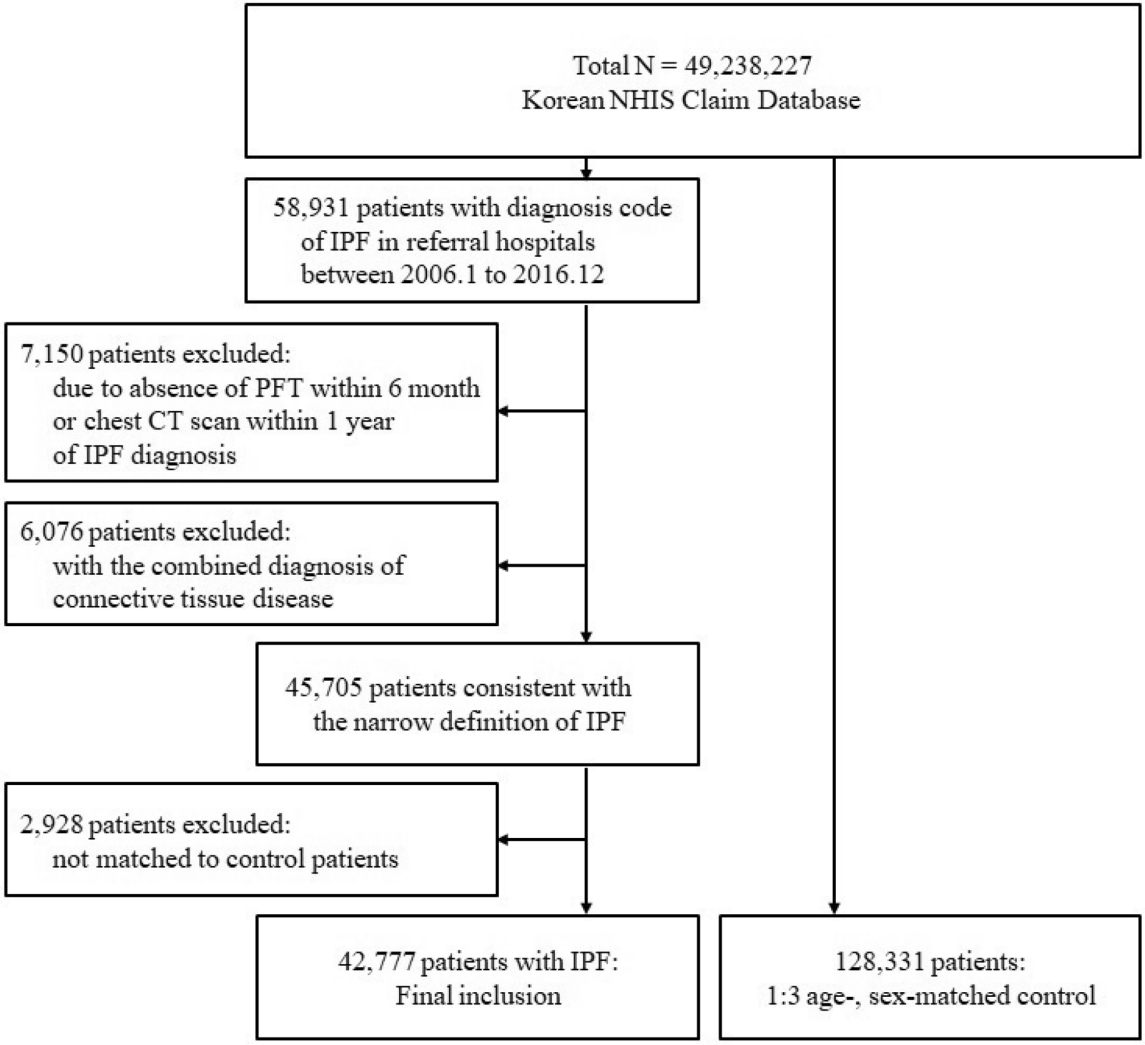
Inclusion flow chart. *NHIS* National Health Insurance Service, *IPF* idiopathic pulmonary fibrosis, *PFT* pulmonary function test, *CT* computed tomography.

From January 2006 to December 2016, 58,931 patients were identified in the NHIS database as having been diagnosed with IPF at secondary or tertiary referral hospitals. The date of IPF diagnosis was defined as the index date of the IPF patients and they were followed thereafter. Patients who did not undergo a pulmonary function test within 6 months or chest CT scan within 1 year of the index date (7150 patients) were excluded from the analysis. Patients who were diagnosed with CTD (6076 patients) were also excluded. The remaining 45,705 patients with IPF were matched with controls according to age and sex at a 1:3 ratio. After matching, 2928 patients with IPF whose matched controls died before the index date were additionally excluded. Finally, this study included 42,777 patients diagnosed with IPF and a 1:3 control group of 128,331 people (Fig. 1).

### Baseline characteristics

The mean age of the IPF patients was 64.6 ± 13.8 years, and 65.4% were male. Compared to the control group, patients in the IPF group more often had ischemic heart disease, cerebrovascular disease, cancer, diabetes mellitus, hypertension, dyslipidemia, and chronic liver disease. Household income and place of residence did not differ between the two groups (Table 1).

**Table 1:** Baseline participant characteristics.

	IPF group	Control group	*p* value
Number of participants (n)	42,777	128,331	
Age, mean ± SD (years)	64.6 ± 13.8	64.6 ± 13.8	> 0.999
Male sex, n (%)	27,971 (65.4)	83,913 (65.4)	> 0.999
**Comorbidities**			
Ischemic heart disease, n (%)	11,592 (27.1)	14,853 (11.6)	< 0.001
Cerebrovascular disease, n (%)	19,342 (45.2)	51,494 (40.1)	< 0.001
Cancer, n (%)	5246 (12.3)	5678 (4.4)	< 0.001
Diabetes mellitus, n (%)	8655 (20.2)	18,303 (14.3)	< 0.001
Hypertension, n (%)	17,530 (41.0)	48,857 (38.1)	< 0.001
Dyslipidemia, n (%)	9676 (22.6)	21,930 (17.1)	< 0.001
Chronic liver disease, n (%)	11,033 (25.8)	25,305 (19.7)	< 0.001
**Household income, n (%)**			0.259
1st quintile	10,623 (24.8)	31,017 (24.2)	
2nd quintile	6547 (15.3)	20,269 (15.8)	
3rd quintile	7078 (16.6)	21,413 (16.7)	
4th quintile	8455 (19.8)	25,313 (19.7)	
5th quintile	10,074 (23.6)	30,319 (23.6)	
**Place of residence, n (%)**			0.207
Urban	23,692 (55.4)	71,525 (55.7)	
Rural	19,085 (44.6)	56,806 (44.3)	

*IPF* idiopathic pulmonary fibrosis, *SD* standard deviation.

### All-cause mortality rate of patients with IPF

The all-cause mortality rate of the patients with IPF was 85.663 per 1000 person-years, which was approximately three times higher than that of the control group (29.525 per 1000 person-years, *p* < 0.001). The mortality rate of male IPF patients was 97.617 per 1000 person-years, which was significantly higher than that of female IPF patients (65.895 per 1000 person-years, *p* < 0.001). The HR of mortality in patients with IPF compared to that in the control group was 2.918 after adjusting for age, sex, presence of comorbidities, household income, and place of residence. The adjusted HRs of male and female IPF patients compared to the control group were 2.885 and 2.989, respectively (Table 2).

**Table 2:** All-cause and cause-specific mortality rates of patients with idiopathic pulmonary fibrosis (IPF).

		Total patients (n)	Death (n)	Follow-up duration (person-year)	Mortality rate per 1000 person-year	^a^Adjusted HR (95% CI)	*p* value
**Total death**							
Total	Control	128,331	25,058	848,706	29.525	1 (Ref.)	
	IPF	42,777	18,905	220,690	85.663	2.918 (2.861–2.976)	< 0.001
Male	Control	83,913	18,153	543,748	33.385	1 (Ref.)	
	IPF	27,971	13,425	137,527	97.617	2.885 (2.819–2.953)	< 0.001
Female	Control	44,418	6905	304,958	22.643	1 (Ref.)	
	IPF	14,806	5480	83,162	65.895	2.989 (2.881–3.101)	< 0.001
**Respiratory cause**							
Total	Control	128,331	3140 (12.5%)	848,706	3.700	1 (Ref.)	
	IPF	42,777	7147 (37.8%)	220,690	32.385	9.837 (9.421–10.271)	< 0.001
Male	Control	83,913	2451 (13.5%)	543,748	4.508	1 (Ref.)	
	IPF	27,971	4828 (36.0%)	137,527	35.106	8.699 (8.273–9.147)	< 0.001
Female	Control	44,418	689 (10.0%)	304,958	2.259	1 (Ref.)	
	IPF	14,806	2319 (42.3%)	83,162	27.885	13.847(12.697–15.101)	< 0.001
**Cancer**							
Total	Control	128,331	6459 (25.8%)	848,706	7.610	1 (Ref.)	
	IPF	42,777	4528 (24.0%)	220,690	20.518	2.381 (2.289–2.477)	< 0.001
Male	Control	83,913	5225 (28.8%)	543,748	9.609	1 (Ref.)	
	IPF	27,971	3719 (27.7%)	137,527	27.042	2.475 (2.369–2.586)	< 0.001
Female	Control	44,418	1234 (17.9%)	304,958	4.046	1 (Ref.)	
	IPF	14,806	809 (14.8%)	83,162	9.728	1.990 (1.814–2.183)	< 0.001
**Cardiovascular cause**							
Total	Control	128,331	1818 (7.3%)	848,706	2.142	1 (Ref.)	
	IPF	42,777	690 (3.6%)	220,690	3.127	1.415 (1.293–1.549)	< 0.001
Male	Control	83,913	1161 (6.4%)	543,748	2.135	1 (Ref.)	
	IPF	27,971	449 (3.3%)	137,527	3.265	1.448 (1.294–1.621)	< 0.001
Female	Control	44,418	657 (9.5%)	304,958	2.154	1 (Ref.)	
	IPF	14,806	241 (4.4%)	83,162	2.898	1.347 (1.157–1.569)	< 0.001
**Suicide**							
Total	Control	128,331	511 (2.0%)	848,706	0.602	1 (Ref.)	
	IPF	42,777	258 (1.4%)	220,690	1.169	2.000 (1.712–2.335)	< 0.001
Male	Control	83,913	443 (2.4%)	543,748	0.815	1 (Ref.)	
	IPF	27,971	212 (1.6%)	137,527	1.542	1.940 (1.637–2.300)	< 0.001
Female	Control	44,418	68 (1.0%)	304,958	0.223	1 (Ref.)	
	IPF	14,806	46 (0.8%)	83,162	0.553	2.355 (1.600–3.467)	< 0.001
**Others**							
Total	Control	128,331	13,130 (52.4%)	848,706	15.471	1 (Ref.)	
	IPF	42,777	6282 (33.2%)	220,690	28.465	1.861 (1.804–1.920)	< 0.001
Male	Control	83,913	8873 (48.9%)	543,748	16.318	1 (Ref.)	
	IPF	27,971	4217 (31.4%)	137,527	30.663	1.856 (1.787–1.928)	< 0.001
Female	Control	44,418	4257 (61.7%)	304,958	13.959	1 (Ref.)	
	IPF	14,806	2065 (37.7%)	83,162	24.831	1.855 (1.757–1.959)	< 0.001

*IPF* idiopathic pulmonary fibrosis, *HR* hazard ratio, *CI* confidence interval, *Ref* reference.

^a^Adjusted by age, sex, ischemic heart disease, stroke, cerebrovascular disease, cancer, diabetes mellitus, hypertension, dyslipidemia, liver disease, household income, and place of residence.

**Figure 2:**
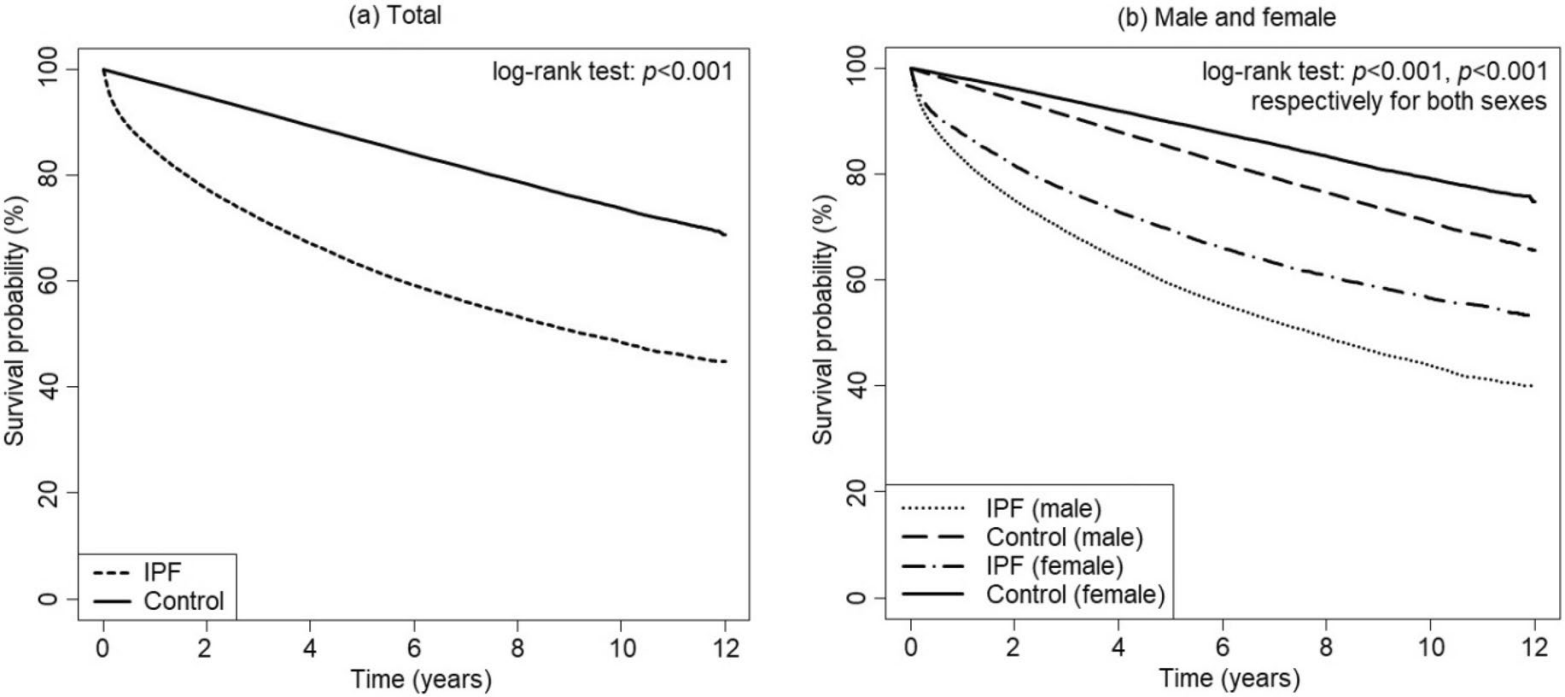
Survival rate of patients with idiopathic pulmonary fibrosis (IPF) compared to control group by Kaplan–Meier curve analysis.

The survival rates of IPF patients were 84.5%, 77.4%, 71.9%, 62.9%, and 48.4% at 1, 2, 3, 5, and 10 years of follow-up, respectively. Kaplan–Meier curves of male and female IPF patients and their controls are shown in Fig. 2.

### Cause-specific mortality rate of patients with IPF

The leading cause of death in IPF patients was respiratory causes, followed by cancer. Among IPF patients, 37.8% (7417 of 18,905) died due to respiratory causes, which was about ten times higher than that in the control group (adjusted HR 9.743, *p* < 0.001). Patients with IPF also showed higher risks of death from cancer, cardiovascular cause, and suicide compared to the control group (adjusted HR 3.007, 1.739, and 2.085, respectively; *p* < 0.001). Respiratory causes of death were more frequently observed in females and cancer was more frequently observed in males (Table 2).

### Standardized mortality ratio of IPF patients

Compared to the expected mortality rate of the general population, the SMR of IPF patients was 4.659 (95% confidence interval 4.593–4.726). Although the mortality rate was higher in the older age group than that in the younger age group, the SMR was lower in the older age group, except for that in patients in their 50 s, which was slightly higher than the SMR in the 40 s in both sexes (Table 3).

**Table 3:** Standardized mortality ratio of patients with idiopathic pulmonary fibrosis (IPF) according to age group.

		Death	Follow-up duration (person-year)	Mortality rate per 1000 person-year	SMR (95% CI)
Total		18,905	220,690	85.7	4.659 (4.593–4.726)
Male	20–29	40	3824	65.4	15.993 (11.037–20.949)
	30–39	51	6848	107.6	6.922 (5.022–8.822)
	40–49	255	14,537	282.6	6.207 (5.445–6.968)
	50–59	1217	27,797	622.2	7.037 (6.641–7.432)
	60–69	3933	44,924	1434.9	6.101 (5.910–6.292)
	70–79	5810	32,354	3926.1	4.574 (4.456–4.691)
	80-	2119	7242	11,215.8	2.609 (2.498–2.720)
Female	20–29	35	2995	40.2	29.030 (19.413–38.648)
	30–39	35	4888	61.6	11.622 (7.772–15.473)
	40–49	112	10,740	110.6	9.427 (7.681–11.173)
	50–59	325	16,291	209.6	9.518 (8.484–10.553)
	60–69	995	20,947	560.7	8.471 (7.945–8.998)
	70–79	2524	20,814	1978.5	6.129 (5.890–6.368)
	80–	1454	6487	8124.8	2.759 (2.617–2.900)

*IPF* idiopathic pulmonary fibrosis, *SMR* standardized mortality ratio, *CI* confidence interval.

### ICU admission before death in patients with IPF

Among the deceased IPF patients, 13.8% were admitted to the ICU before death. The proportion of patients with ICU admission before death was higher in male patients than in female patients (14.2% vs. 12.7%, *p* = 0.006).

## Discussion

This nationwide general population-based study showed that Korean IPF patients had significantly higher all-cause and cause-specific mortality rates compared to those in the control group without IPF. To our knowledge, this is the first nationwide, large-scale, general population-based study on this subject in Asia. Previous two studies from Japan and Taiwan were either with only a small number of 553 IPF patients^11^ or used a database representing only 10% of all insured Taiwanese patients, and showed a much lower IPF incidence rate^12^ compared to other epidemiologic studies of IPF.

In this study, the survival rates of IPF patients were 84.5%, 77.4%, and 71.9% at 1, 2, and 3 years after diagnosis, respectively. These results are comparable to those of a multicenter cohort study in Korea that reported 1-, 2-, and 3-year the survival rates of 82.7%, 75.7%, and 70.3%, respectively, among 832 IPF patients^14^. A population-based study from Canada reported a survival rate of 79.0%, 69.5%, and 63.2% at 1, 2, and 3 years after diagnosis, respectively^7^. The differences in survival rate are compatible with the results of a recent study in the United States, which observed a lower mortality rate among Asian patients with IPF than that of Caucasian or Hispanic IPF patients^6^.

The all-cause mortality rate of IPF patients was 97.617 per 1000 person-years in men and 65.895 in women in the present study, which was three times higher than that of the control group. Population-based studies reporting the mortality rate of IPF patients consistently showed higher mortality in male patients than that in female patients^2–4,6,9^, except for a study in Brazil that reported similar mortality between sexes^20^. A recent cohort study reported that the presence of cough was associated with higher mortality only in men but not in women^21^; however, the cause of sex differences in IPF mortality remains unknown.

According to the SMR calculated in our study, Korean IPF patients had a 4.7-fold higher risk of mortality than that in the general population. In contrast to the mortality rate of IPF patients which increased with age, the SMR of IPF patients decreased with age. Similar trends have been observed in studies investigating other diseases^22,23^. Although the absolute mortality rate was higher in older patients, the impact of IPF on mortality was higher in younger patients because the expected mortality of the general population at a younger age is very low. To our knowledge, ours is one of the few studies to report the SMR of IPF patients.

Although respiratory causes were the consistent leading cause of death among IPF patients, the proportion of patients who died due to respiratory causes was lower and that of cancer was higher than in reported in previous population-based studies^2,6,11^. As mentioned above, Asian patients with IPF have longer expected survival compared to that in Caucasian patients, which makes them more prone to cancer development during their lives. In Korea, the National Cancer Screening Program allows cost-free screening of stomach, breast, cervix, liver, and colon cancer for almost all Korean citizens^24^. Moreover, the risk of cancer development in patients with IPF is more than twice that in people without IPF^25^. A significant number of patients diagnosed with cancer through screening may also have been diagnosed with early IPF, which explains the high prevalence of cancer in our study participants. Patients with comorbidities such as IPF may not as receive aggressive cancer treatments as do those without IPF, which might explain the high rate of death by cancer in our study. A cohort study showed higher IPF-specific mortality in females, although the overall mortality was higher in males^26^, which was consistent with our findings.

Among IPF patients who were admitted to the hospital, more than 10% were mechanically ventilated; however, their mortality rate was sevenfold higher than that of patients not mechanically ventilated, with considerable additional cost^27^. When mechanically ventilated, the reported mortality rates of IPF patients are as high as 50–94%^17,28,29^. Thus, the initiation of mechanical ventilation should be recommended in selected IPF patients such as those who are candidates for lung transplantation or patients with reversible causes of respiratory failure^18,30^. In our study, 13.8% of IPF patients received ICU care before death, and the proportion of patients with ICU admission was higher in men. Previous studies reported that 18.1% of the general population of Korea died in the ICU^31^, compared to 22.4% of the general population of the United States^32^, with 29.0% of Medicare beneficiaries receiving ICU care during the last 30 days of their lives^33^. As expected, the proportion of deaths in the ICU of IPF patients in our study was much lower than that reported in the general population.

Our study has several limitations. First, we used the claims data of the NHIS, which limited the diagnostic accuracy of IPF. To overcome this limitation, we used not only the disease code but also several additional criteria as described in the method section. Second, death certificate data were obtained to evaluate the cause of death. Although errors in the cause and/or manner of death recorded on the certificates are reportedly high^34^, the major error is an overrepresentation of nonspecific cardiovascular events as the cause of death^35^. In our study, the major cause of death was respiratory causes, followed by cancer, with only 3.6% attributed to cardiovascular causes. Third, we only described the use of ICU facilities within 30 days before the date of death but could not evaluate the reason and appropriateness of ICU admission or whether the reason for ICU admission was correspondent to the cause of death. Fourth, information on the specifics of the cause of death or the presence of acute exacerbation could not be obtained due to the nature of the database.

In conclusion, IPF patients showed a significantly higher mortality rate compared to that in the general population. The leading cause of death in IPF patients was respiratory causes, followed by cancer. The proportion of patients requiring ICU services before death in IPF patients was lower than that in the general population reported in previous studies.

## Data Availability

Data are accessible from NHIS database, but the access to data used in this study is only available for the researchers who have applied for and have been granted. Further information is available in online homepage of National Health Insurance Sharing Service (https://nhiss.nhis.or.kr).
