# Publisher Correction: All-cause mortality of patients with idiopathic pulmonary fibrosis: a nationwide population-based cohort study in Korea

**DOI:** 10.1038/s41598-021-95929-0

**Published:** 2021-08-05

**Authors:** Sung Jun Ko, Sun Mi Choi, Kyung-Do Han, Chang-Hoon Lee, Jinwoo Lee

**Affiliations:** 1 Department of Internal Medicine, Wonkwang University Sanbon Hospital, Gunpo, Republic of Korea; 2 Division of Pulmonary and Critical Care Medicine, Department of Internal Medicine, Seoul National University College of Medicine, Seoul National University Hospital, 101 Daehak-Ro, Jongno-Gu, Seoul, 03080 Republic of Korea; 3 Department of Biostatistics, College of Medicine, The Catholic University of Korea, Seoul, Republic of Korea

Correction to: *Scientific Reports* https://doi.org/10.1038/s41598-021-94655-x, published online 26 July 2021

The original version of this Article contained an error in the spelling of the author Sung Jun Ko which was incorrectly given as Sung Ko. The original Article has been corrected.

